# Evaluation of leakage resistance improvement in transconjunctival sutureless vitrectomy sclerotomies closed with adhesives. an experimental study

**DOI:** 10.1038/s41433-019-0651-4

**Published:** 2019-11-04

**Authors:** Lorenzo López-Guajardo, Javier Benítez-Herreros, Juan Donate-López, Valeria Opazo-Toro

**Affiliations:** 1 Ophthalmology Department, Hospital Clínico “San Carlos”, Madrid, Spain; 2 Ophthalmology Department, Hospital Universitario “Príncipe de Asturias”. Alcalá de Henares, Madrid, Spain; 3 Universidad Complutense, Madrid, Spain

**Keywords:** Outcomes research, Surgery

## Abstract

**Background:**

The purpose of this paper is to study the utility of adhesives (artificial-cyanoacrylate and biological-fibrin glue) for improving transconjunctival sutureless vitrectomy (TSV) sclerotomy closure competency.

**Methods:**

Experimental and observer-masked study in which after performing TSV in cadaveric pig eyes, different adhesives were tested on sclerotomy entrances in order to determine if they improved closure competency in face of progressive intraocular pressure increase. In 76 eyes cyanoacrylate-treated sclerotomies were compared with sclerotomies in which no additional manoeuvre to aid closing was performed; in 76 eyes fibrin glue with no manoeuvre; and in the last 76 eyes, cyanoacrylate-treated sclerotomies were compared with fibrin glue-treated sclerotomies.

**Results:**

A total of 228 eyes had a 23-gauge TSV performed. Both cyanoacrylate and fibrin glue treated sclerotomies achieved higher mean opening pressures when compared with nontreated sclerotomies in the same eye (*p* < 0.002). When cyanoacrylate was compared with biological adhesive in the same eye, no statistically significant differences were obtained (*p* = 0.216).

**Discussions:**

This experimental study provides support for the possible role of adhesives in improving TSV sclerotomy closure competency in clinical practice.

## Introduction

Vitrectomy is one of the most frequently performed ophthalmic surgical procedures (excluding cataract surgery). All vitrectomies require an access to the vitreous cavity that is performed through a sclerotomy of variable diameter. In the past decades, three-port vitrectomy has become practically the sole surgical technique, which implies thousands of sclerotomies performed every year around the world. Actual trends imply a reduction in sclerotomy diameter, in order to achieve a “transconjunctival sutureless vitrectomy” (TSV). TSV was developed as a technique that could result in a self-sealing sclerotomy without suturing due to the small gauge used; but this has not been the case for all sclerotomies. Surgical practice has demonstrated that not all sclerotomies achieve competent postoperative closure once the instruments are removed, even after performing oblique sclerotomy construction [1]. Authors refer a variable percentage of sclerotomies (between 0 and 38.5%) that need an additional manoeuvre in order to achieve correct closure [2–5].

This data reflects the importance of every aspect of sclerotomy study, including any manoeuvre that improves closure rate. Incompetent sclerotomies can transform a successful surgery into a hypotonus complicated eye (increased risk of endophthalmitis, choroidal detachment, and premature loss of tamponade agents, etc.).

Biological and artificial adhesives have been used in many surgical settings, and have been already used in ophthalmology [6, 7]. They have proven to be useful and simple to apply. Although cyanoacrylate has a higher tensile strength than fibrin adhesives, it has been associated with histotoxicity. Fibrin is biodegradable and induces less toxic and inflammatory reactions [8]. The use of adhesives, as an alternative to commonly used sutures for achieving correct sclerotomy closure, can be more adequate in settings such as thin or staphylomatous scleras or reoperated eyes with same sclerotomy localization; and can diminish postoperative astigmatism and patient discomfort. Both biological (fibrin sealant) and artificial (cyanoacrylate) tissue adhesives were tested in this study.

With this data in mind, we decided to perform this study. The use of a cadaveric animal model allowed us to test mechanical closure capabilities in extreme pressure situations, which we could not have performed in real clinical settings.

## Material and Methods

This experimental and observer-masked study included 234 pig eyes (Sus scrofa domesticus species; 57–81 kg). Exclusion criteria were the presence of scleral alterations and intraoperative complications (retinal detachment or lens dislocation that could occlude sclerotomies), as well as media opacities significant to obstruct surgery. Finally, 228 eyes were included and divided into three groups of 76 eyes. Our first 20 cases revealed a size effect of 30%, which gave us an estimate sample size of 42 eyes (type I error: 0.05; power: 80%), but due to the unlimited source of eyes, we included 76. All eyes were obtained 3 h after pig sacrificed in a regulated slaughterhouse and were kept in cold storage at 4 °C before use, with the aim of preserving the mechanical properties of all ocular tissues [9]. No statements for the use of animals in ophthalmic research were thus applicable.

**Fig. 1:**
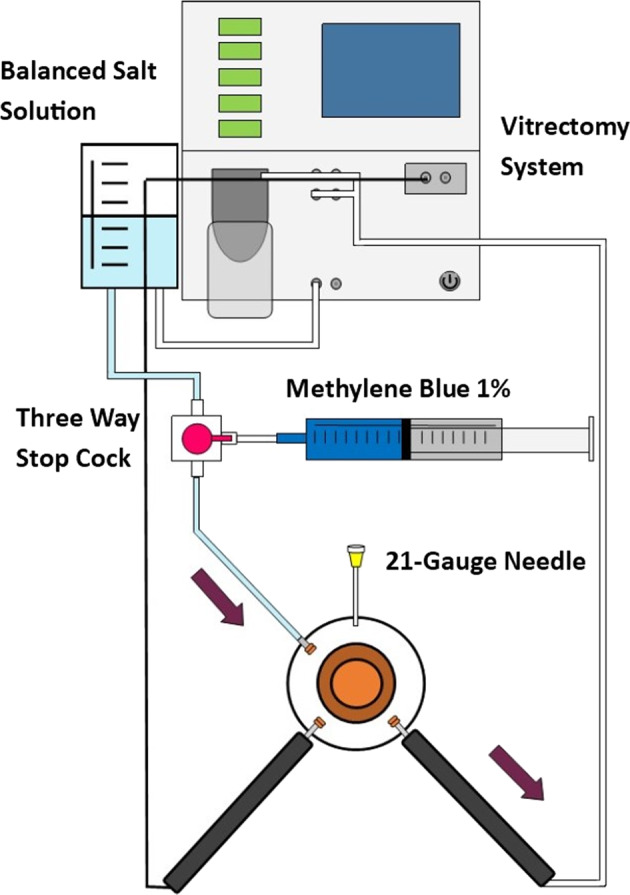
Schematic representation of the experimental setting. The three way stop clock allows for entering blue dyed balanced salt solution (BSS) after completing 23 g vitrectomy into the vitreous cavity

**Fig. 2:**
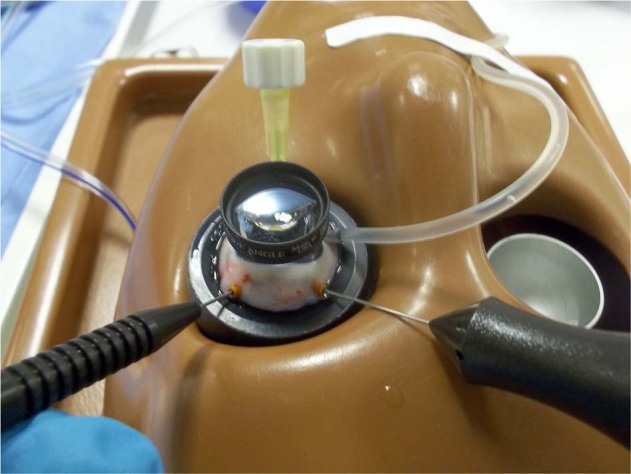
A 23-gauge TSV is performed in each eye, with the 23 gauge needle at 6 o’clock closed

**Fig. 3:**
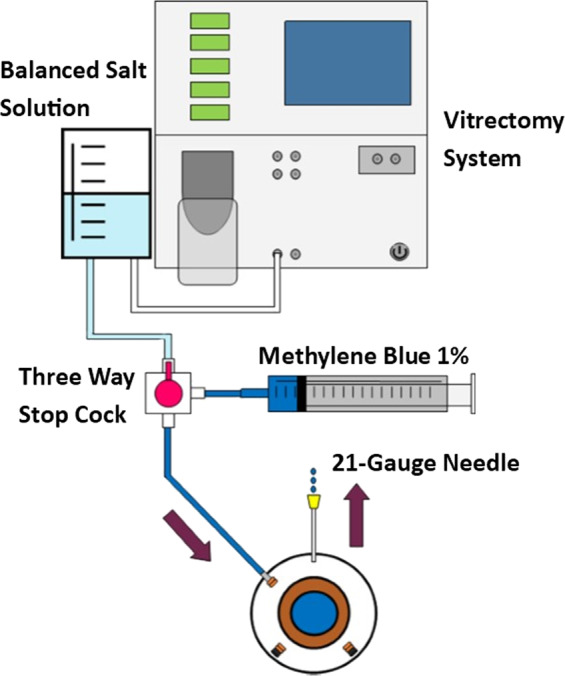
After vitrectomy, blue-dyed solution is introduced in the vitreous cavity through the infusion line and exchanged with clear BSS evacuated through the 23 gauge needle

**Fig. 4:**
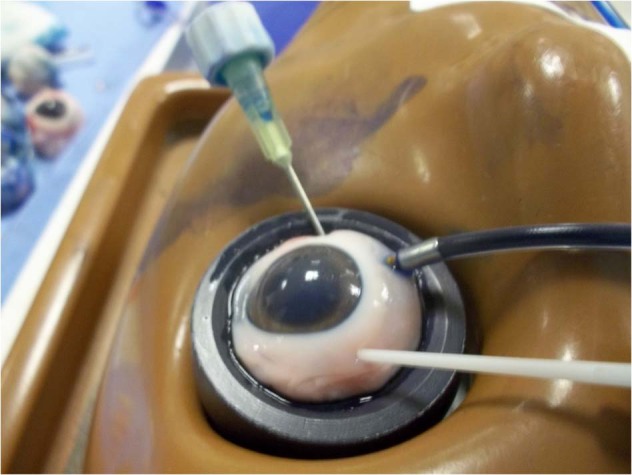
Once blue solution fills the eye, adhesive is applied to one of the sclerotomies before progressive increase of intraocular pressure

To compare the closure resistance observed in sclerotomies sealed with cyanoacrylate glue (Histoacryl glue; Aesculap AG, Tuttlingen, Germany), sclerotomies closed with the aid of biological adhesive (Tisseel, Baxter AG Industries, Viena, Austria) and sclerotomies closed in the usual manner (no additional manoeuvre), under progressive increases in IOP, we used a validated experimental model of vitrectomized pig eye [10] (Fig. 1). A 23-gauge TSV was performed in each eye. Sclerotomies were performed using an oblique incisional technique 4 mm from the limbus. The globe was penetrated tangentially with the trocar-cannula at an angle of ~30°, parallel to the corneal limbus, all the way of the bevelled trocar up to the beginning of the cannula. Finally, the penetrating direction was modified to a perpendicular orientation (pointing to the centre of the globe) for the rest of the trocar up to the collar of the microcannula [1]. We also inserted a closed 21-gauge needle into vitreous cavity at 6 o'clock (Fig. 2). Once each 23-gauge vitrectomy was completed (Accurus; Alcon Laboratories, Fort Worth, TX), intraocular BSS was exchanged with 1% methylene blue solution introduced through the infusion line and allowing clear BSS to drain through the 21-gauge needle opened to exterior (Fig. 3). Then, both superior cannulas were extracted at 5 mmHg, so no dye leaked through the sclerotomy. After that, cyanoacrylate, biological glue or alternatively, no closure manoeuvre was performed on each superior sclerotomy. Adhesives were applied on the scleral and conjunctival edges of the chosen superior incision sites (Fig. 4).

With the aim of avoiding the influence that the superior sclerotomy use (vitreous cutter or illumination probe) may have on the postoperative sclerotomy closure competency, the two closure methods were applied on both sclerotomy sites according to their use in an equal manner. In that way, in 76 randomly chosen eyes, we compared cyanoacrylate versus no closure manoeuvre; in 38 of those eyes cyanoacrylate was applied only on the incisions used by the vitreous cutter whereas no manoeuvre was performed on the sclerotomies used by the light probe; in the other 38 globes, cyanoacrylate was applied on the sclerotomies employed for the entrance of the light pipe (no manoeuvre on the vitreous cutter incision). In other 76 randomly chosen eyes, we used both cyanoacrylate and biological adhesive closures; in 38 of them, cyanoacrylate and biological adhesive were applied on the vitreous cutter and the light pipe incisions respectively; in the other 38 globes, the entrances used by the illumination were closed with the aid of cyanoacrylate and the vitreous cutter sclerotomies were closed using biological adhesive. In the last 76 eyes, we compared biological adhesive versus no additional closure manoeuvre; in 38 eyes biological glue was applied only on the wounds used by the vitreous cutter (no manoeuvre was performed on the sclerotomies used by the light probe); in the other 38 globes, biological adhesive was only applied on the sclerotomies employed for the entrance of the light pipe (no manoeuvre on the vitreous cutter incisions).

**Fig. 5:**
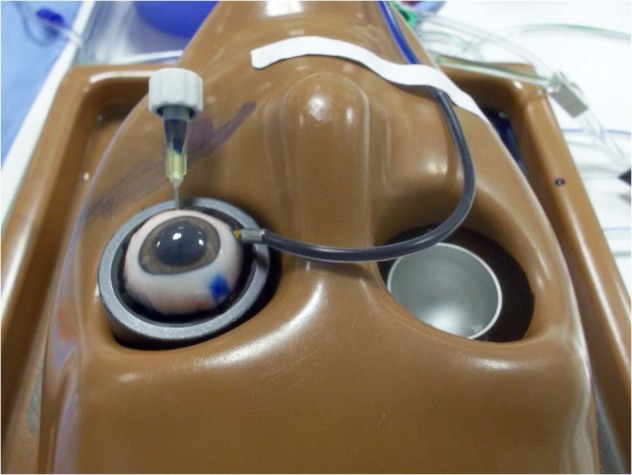
Blue solution flows out of the leaking sclerotomy

After performing the closure manoeuvre, IOP was gradually increased in 5 mmHg steps using the vented gas forced infusion of the vitrectomy system (VGFI; Accurus; Alcon Laboratories, TX) until one of the superior sclerotomies opened, allowing internal ocular blue-dyed solution to escape (Fig. 5). This was readily detected by the appearance of a blue subconjunctival bleb at the level of the sclerotomy or directly by a blue fluid escape flow. In some cases when 120 mmHg was reached, no sclerotomy had yet opened, so pressure was increased by the surgeon’s thumb (LLG), which pressed on the centre of the cornea until one of the superior sclerotomies opened. Because of this, pressure values >120 mmHg could not be precisely quantified and therefore, an arbitrary value was given for statistical purposes. All results in a database (which sclerotomy was the first to leak intraocular fluid—cyanoacrylate, biological glue or non-treatment—and which IOP level was reached).

For all statistical tests, 5% was considered the significance level (*p* < 0.05). The frequency of sclerotomies which leaked first depending on the closure technique was studied using the Chi square test corrected for continuity. To compare the differences in IOP values at which different groups of sclerotomies leaked, we used the nonparametric Mann–Whitney U test; pressures higher than 120 mmHg could not be quantified consistently and thus, it was not possible to use a *t*-test.

## Results

Six eyes had to be excluded from the study due to intraoperative complications (retinal breaks or retinal detachments) or media opacities (cataract/cornea) that did not allow satisfactory vitrectomy performance at authors discretion. Overall, 228 eyes were finally included. Postmortem vitreous was clear in all globes. Most eyes did not present posterior vitreous detachment; however, for studying the sclerotomy closure resistance, we did not need to extract posterior hyaloid but just enough core and anterior vitreous to allow BSS to flow freely through the cannulas after removing the instruments.

When we analysed the sample of 76 randomly chosen eyes in which cyanoacrylate was applied on one of the superior sclerotomies and no additional closure manoeuvre was performed on the other incision, in 26 of them (34.2%; 95% CI: 24.0–46.1%) the sclerotomy sealed with cyanoacrylate opened first when IOP was increased (*p* = 0.001; Chi-Square test); when we studied the IOP values, treated sclerotomies opened at a significantly higher pressure level than nontreated incisions (*p* < 0.001; Mann–Whitney U test). When we evaluated the 76 globes in which cyanoacrylate or biological glue were applied on superior incisions, 42 of the leaking wounds (55.3%; 95% CI: 43.5–66.5%) were the ones treated with biological glue (*p* = 0.256; Chi-Square test); no statistically differences were found when we analysed IOP levels in which sclerotomies opened (*p* = 0.216; Mann–Whitney U test). Finally, when studying the sample of eyes in which biological adhesive was compared with sclerotomies with no additional closure manoeuvre, in 28 of 76 eyes (36.8%; 95% CI: 26.3–48.7%) the sclerotomy treated with glue opened first. (*p* = 0.002; Chi-Square test); when we analysed the IOP values, fibrin-treated sclerotomies opened at a significantly higher-pressure level (*p* < 0.001; Mann–Whitney U test).

## Discussion

This work has been done on a cadaveric animal model. We believe it is the most adequate model, as cadaveric human eyes are difficult to obtain, and we needed an important number of eyes. Timing from death to operation was important in order to avoid as much as possible tissue decay, which would probably alter the mechanical properties. This can only be possible with eyes coming from regulated slaughter. Pig eyeball has important structural resemblances with human eyes, and our animal model has been already tested for studying different aspects of sclerotomy closure after microincisional vitrectomy [11–14]. Porcine sclera holds important similarities with human eyes regarding histological characteristics, collagen bundle organization and water content; however, we must assume a greater thickness in pig sclera with respect to human [15]. Given this fact, we do not aim to obtain quantitative results which could be extrapolated to human eyes. The purpose of the study is just to analyse if the application of biological or synthetic glue could have any effect on incisional closure competence.

Mechanical forces are the main mechanism for sclerotomy closure during the immediate postoperative period, which is when most complications related to sclerotomy incompetence occur (hypotony, choroidal detachment, endophthalmitis [16]-although it must be stated that other studies did not find differences with 20-gauge vitrectomy [17]. Active healing processes that develop later are obviously not addressed in our cadaveric model.

With both the biological adhesive and cyanoacrylate, we were able to obtain an adequate adhesion, results also obtained in rabbit eye [18]. We must mention cadaveric porcine conjunctiva was less hydrated and thus thinner than the human living conjunctiva we find in surgical room, but we have been able to reproduce this manoeuvre in regular surgical cases in which we were incapable to obtain proper sclerotomy closure (unpublished data). In these cases we have only used fibrin glue (Tisseel), which we believe causes less irritation than cyanoacrylate. Other authors also have been able to achieve it in clinical setting [19–24].

Absolute pressure values at which the sclerotomy begins leaking need not be considered. As we see, in our model, sclerotomy incompetence begins when pressure is raised to 35 mmHg, and no spontaneous immediate leaking occurs, as we sometimes see in regular surgery (this fact could be due to the relative thicker porcine sclera when compared with human as mentioned earlier). Thus, it is the relative values when comparing sclerotomies that were closed with adhesives with sclerotomies without adhesives that are relevant. It has been published that certain situations, such as rubbing the eye, can raise intraocular pressure up to 310 mmHg from basal levels [25]. This situation can only be studied using a nonhuman model, as is our case. We were only able to quantify pressures as high as 120 mmHg due to limitations of Alcon Accurus VGFI system, but with digital pressure we surely reached values as high as the reported values with eye rubbing.

As we know, the most extended method for achieving a correct closure of a leaking sclerotomy in the immediate postoperative period is using sutures. This solution produces patient discomfort, induces astigmatism, and requires suture removal in the postoperative period. In cases of very thin sclera, surgeons have even difficulties in performing the stitch without creating tears in peri-sclerotomy sclera when tensioning the knot. In reoperations, when sclerotomies are placed very closely together, a similar problem appears. With the proper limitations of translational research, this work can provide support for a different option of achieving correct sclerotomy closure, which avoids in part the previously related drawbacks [19, 20], but that has a cost limitation that must be evaluated for our health systems in specific clinical settings.

### Summary

#### What was known before

- Transconjunctival sutureless vitrectomy, specially 23 gauge, faces occasional sclerotomy closure incompetence
- Postoperative sclerotomy leakage increases risk of hypotony, endophthalmitis, and other related complications
- Most frequent method for solving this problem is placing sutures in the sclerotomy entrance
- Scleral sutures cause discomfort, postoperative astigmatism and may be difficult to perform in thin scleras

#### What this study adds

- With limitations inherent to translational research
- This work can provide support for the use of adhesives in achieving correct sclerotomy closure
- This closure option avoids inducing suture-related astigmatism and difficulties of suturing thin scleras
- Has a cost limitation that must be evaluated in specific clinical settings.
